# The transcriptional network of *WRKY53* in cereals links oxidative responses to biotic and abiotic stress inputs

**DOI:** 10.1007/s10142-014-0374-3

**Published:** 2014-04-29

**Authors:** Leon Van Eck, Rebecca M. Davidson, Shuchi Wu, Bingyu Y. Zhao, Anna-Maria Botha, Jan E. Leach, Nora L. V. Lapitan

**Affiliations:** 1 Department of Soil and Crop Sciences, Colorado State University, Fort Collins, CO 80523 USA; 2 Department of Genetics, Stellenbosch University, Stellenbosch, Western Cape 7600 South Africa; 3 Department of Bioagricultural Sciences and Pest Management, Colorado State University, Fort Collins, CO 80523 USA; 4 Integrated Center for Genes, Environment & Health, National Jewish Health, Denver, CO 80206 USA; 5 Department of Horticulture, Virginia Tech, Blacksburg, VA 24061 USA

**Keywords:** *Triticum aestivum*, *Oryza sativa*, Plant disease resistance, WRKY transcription factors, Gene regulation, Protein–DNA interactions

## Abstract

The transcription factor *WRKY53* is expressed during biotic and abiotic stress responses in cereals, but little is currently known about its regulation, structure and downstream targets. We sequenced the wheat ortholog *TaWRKY53* and its promoter region, which revealed extensive similarity in gene architecture and *cis*-acting regulatory elements to the rice ortholog *OsWRKY53*, including the presence of stress-responsive abscisic acid-responsive elements (ABRE) motifs and GCC-boxes. Four proteins interacted with the *WRKY53* promoter in yeast one-hybrid assays, suggesting that this gene can receive inputs from diverse stress-related pathways such as calcium signalling and senescence, and environmental cues such as drought and ultraviolet radiation. The Ser/Thr receptor kinase *ORK10/LRK10* and the apoplastic peroxidase *POC1* are two downstream targets for regulation by the WRKY53 transcription factor, predicted based on the presence of W-box motifs in their promoters and coregulation with *WRKY53*, and verified by electrophoretic mobility shift assay (EMSA). Both *ORK10/LRK10* and *POC1* are upregulated during cereal responses to pathogens and aphids and important components of the oxidative burst during the hypersensitive response. Taken with our yeast two-hybrid assay which identified a strong protein–protein interaction between microsomal glutathione *S*-transferase 3 and WRKY53, this implies that the *WRKY53* transcriptional network regulates oxidative responses to a wide array of stresses.

**Electronic supplementary material:**

The online version of this article (doi:10.1007/s10142-014-0374-3) contains supplementary material, which is available to authorized users.

## Introduction

*WRKY53* is a WRKY transcription factor integral to several biotic and abiotic stress resistance responses in cereals such as wheat (*Triticum aestivum* L.) and rice (*Oryza sativa* L.). The rice ortholog, *OsWRKY53*, is expressed in roots and leaves and is inducible by drought stress and chitinous elicitors (Akimoto-Tomiyama et al. 2003; Ramamoorthy et al. 2008). The wheat ortholog, *TaWRKY53*, is induced during leaf senescence (Wu et al. 2008) and infestation by the Russian wheat aphid, *Diuraphis noxia* Kurdjumov (Botha et al. 2010; Smith et al 2010). Overexpression of *OsWRKY53* induces pathogenesis-related (PR) protein expression and greatly reduces symptoms of infection by the rice blast fungus, *Magnaporthe oryzae* (Hebert) Barr (Chujo et al. 2007; Marcel et al. 2010), whereas silencing of *TaWRKY53* results in suppression of the oxidative burst and increased aphid susceptibility (Van Eck et al. 2010).

The recent systematic census and phylogenetic analysis of 92 *WRKY* transcription factors in wheat by Zhu et al. (2013) has greatly expanded our knowledge of this transcription factor family and its role in stress regulation in this species. However, apart from some EST information (Wu et al. 2008), almost nothing is known about the structure and function of *TaWRKY53*. Upstream and downstream components of the *WRKY53* transcriptional network have also not been identified in either wheat or rice. Considering the prominent role of *WRKY53* in biotic (Chujo et al. 2007; Van Eck et al. 2010) and abiotic stress (Ramamoorthy et al. 2008; Wu et al. 2008) and thus its potential as a target for plant improvement, we characterized the structure of *TaWRKY53* and its promoter region and then identified upstream and downstream genetic components of the *WRKY53* transcriptional network of the cereals wheat and rice.

## Experimental procedures

### *TaWRKY53* promoter isolation and characterization

Wheat genomic DNA isolated from the *D. noxia*-resistant cv. ‘Gamtoos-R’ (GR) (Van Eck et al. 2010) was employed in genome walking using the GenomeWalker Universal Kit (Clontech, Mountain View, CA, USA) according to the manufacturer’s instructions. Nested primers specific to the 5′ end of the *TaWRKY53* coding sequence (CDS) were designed based on accession EF368357, a *WRKY53* cDNA clone isolated from hexaploid wheat (*T. aestivum* L.) cv. ‘Nongda 3338’ (Wu et al. 2008). Primary and secondary digest library amplifications were performed using LongAmp Taq (New England Biolabs, Ipswich, MA, USA) and nested adaptor-specific primers and gene-specific primers TaWRKY53_GSP1 and TaWRKY53_GSP2 (Table 1). The following cycling parameters were used: initial denaturation at 94 °C for 30 s; 7 amplification cycles consisting of denaturation at 94 °C for 25 s, annealing and extension at 72 °C for 3 min; 32 amplification cycles consisting of denaturation at 94 °C for 25 s, annealing and extension at 67 °C for 3 min; final extension at 67 °C for 7 min. The presence of amplification products was verified by agarose gel electrophoresis. Individual amplicons were gel purified, cloned into the pGEM-T Easy vector (Promega, Madison, WI, USA) and sequenced. Sequence reads obtained from genome walking were assembled into contigs using Geneious Pro 5.4 (Drummond et al. 2011). Promoter characterization was performed using a combination of the *cis*-acting regulatory element databases PLACE (Higo et al. 1999) and PlantCARE (Lescot et al. 2002), and manual scans for the presence of putative W-boxes using the (C/T)TGAC(C/T) consensus sequence.

**Table 1:** Primers for the characterization of *WRKY53* in wheat

Purpose	Primer ID	Primer sequence
Genome walking	TaWRKY53_GSP1 TaWRKY53_GSP2	CGCCAGACCCTGATAGAAGCTCAGTCAAGG AAGGAGGACATGGCGATCGACGCGACGGAA
Full-length clones	TaWRKY53_CDS_fwd TaWRKY53_CDS_rvs	CCCTGCTCCTCCCGTCGCTC CGTGGACCCACATGTAAACGCCA
Protein expression	TaWRKY53exp_fwd TaWRKY53exp_rvs	ATGTCCTCCTCCACGGGGAGCTTGGACC GCCGCGGCCTAGCCTGCCTAGCTAGCAG

### *TaWRKY53* CDS and gene model

The CDS of *TaWRKY53* was amplified out of cDNA and genomic DNA from GR wheat with primers TaWRKY53_CDS_fwd and TaWRKY53_CDS_rvs (Table 1). The following cycling parameters were used: initial denaturation at 94 °C for 1 min; 37 amplification cycles consisting of denaturation at 94 °C for 20 s, annealing at 60 °C for 20 s, extension at 65 °C for 1:40; final extension at 65 °C for 7 min. PCR products were cloned into the pGEM-T Easy vector and sequenced. Sequences were assembled and aligned with GenBank accessions EF368357 and EF368364 to confirm their identity.

### *TaWRKY53* promoter DNA–protein interaction assays

Proteins interacting with the *TaWRKY53* promoter were identified in yeast one-hybrid assays using Gateway-based DNA bait and protein expression prey vectors (Deplancke et al. 2004). The 1.2-kb promoter region of *TaWRKY53* was amplified in three segments, using PCR primers attB4-Pw53–400_fwd and attB1R-Pw53–400_rvs, attB4-Pw53–800_fwd and attB1R-Pw53–800_rvs, and attB4-Pw53–1200_fwd and attB1R-Pw53–1200_rvs (Table 4) to generate −400:P_*W53*_, −800:P_*W53*_ and −1200:P_*W53*_ with added Gateway *attB* transposition sites. The following cycling parameters were used: initial denaturation at 94 °C for 2 min; 40 amplification cycles consisting of denaturation at 94 °C for 30 s, annealing at 63 °C for 30 s and extension at 65 °C for 50 s; final extension at 65 °C for 10 min. The *attB* PCR products were gel purified, recombined with the pDONR P4-P1R vector (Invitrogen, Carlsbad, CA, USA) in a BP clonase reaction, transformed into chemically competent DH5α *Escherichia coli*, and selected for on LB media containing 20 μg mL^−1^ kanamycin. Recombinant entry clones were isolated and recombined with the pDEST-HIS3 destination vector in separate LR clonase reactions to form three expression clones, which were selected for on LB media containing 100 μg mL^−1^ ampicillin. All expression clones were sequenced to verify insert identity. YM4271 yeast cells were transformed with the corresponding pDEST-HIS3 expression clones to generate three distinct DNA bait strains placing the *HIS3* reporter gene under the control of the −400:P_*W53*_, −800:P_*W53*_ or −1200:P_*W53*_ promoter segments. pDEST-HIS3 expression clones were linearized with *Xho*I restriction endonuclease prior to transformation to assist chromosomal integration of the bait constructs at the YM4271 *his3-200* locus. Recombinant clones were selected on SD/–His/–Ura double dropout (DDO) media. To test autoactivation of the *HIS3* reporter gene and cytotoxicity of the clones, 20 yeast colonies from each double bait strain were replica plated onto SD/–His/–Ura media supplemented with 0, 25, 50, 75 or 100 mM 3-amino–1,2,4-triazole (3-AT) and colony growth was monitored. Colonies that exhibited minimal growth at the lowest possible 3-AT concentration were selected as suitable DNA bait strains for yeast one-hybrid assays. All colonies derived from −800:P_*W53*_ and −1200:P_*W53*_ exhibited high levels of autoactivation in the yeast one-hybrid system; therefore, these promoter elements were not further analyzed. The protein expression prey library consisted of pACTGW-attR prey vectors with in-frame N-terminal fusions of activation domain (AD) to a previously constructed rice biotic stress cDNA library. This library was created from *O. sativa* ssp. *japonica* cv. ‘Nipponbare’ inoculated with either of the two bacterial pathogens *Xanthomonas oryzae* pv. *oryzae* and *X. oryzae* pv. *oryzicola* and incubated for varying lengths of time before mRNA isolation (Niño-Liu et al. 2005). Since aphid feeding induces plant responses that overlap with those induced by fungal pathogen attack (Botha et al. 2005; Kaloshian and Walling 2005; Moran and Thompson 2001; Rodriguez and Bos 2013), and since *WRKY53* is involved in both aphid and pathogen resistance responses (Chujo et al. 2007; Van Eck et al. 2010), this biotic stress-induced library could be exploited in our yeast-hybrid analysis to find interactors involved in diverse biotic stress responses. The −400:P_*W53*_ DNA bait strain was transformed with the prey vector library and selected for on SD/–His/–Leu/–Ura triple dropout (TDO) media supplemented with 60 mM 3-AT. AD vector plasmids were rescued from yeast clones showing positive interactions, subcloned into DH5α *E. coli* in order to obtain a higher yield and tested for the presence of cDNA inserts by PCR with AD_fwd and AD_rvs primers (Table 4) before being sequenced.

### TaWRKY53 protein–protein interaction assays

Proteins interacting with TaWRKY53 were identified in yeast two-hybrid assays using Gateway-based bait and prey vectors expressing the *Saccharomyces cerevisiae* GAL4 binding domain (BD) and activation domain (AD), respectively (Nakayama et al. 2002). The same biotic stress-induced pACTGW-attR prey vector library was utilized as described for yeast one-hybrid assays. The pASGW-attR bait vector consisted of an in-frame N-terminal fusion of BD to a truncated version of the *TaWRKY53* coding sequence lacking the first 180 amino acids to prevent autoactivation (Lai et al. 2011). A clone of *TaWRKY53* was amplified from GR cDNA with primers attB1-tW53_fwd and attB2-W53_rvs (Table 4) and *attB* sites attached to either end via PCR. The following cycling parameters were used: initial denaturation at 94 °C for 30 s; 35 amplification cycles consisting of denaturation at 94 °C for 20 s, annealing at 60 °C for 20 s, extension at 64 °C for 1:40; final extension at 64 °C for 10 min. The resulting 884 bp *attB* PCR fragment was cloned into the pDONR 221 donor vector (Invitrogen) via a BP clonase transposition reaction, forming an entry clone, which was transformed into competent DH5α cells and selected for on LB media containing 20 μg mL^−1^ kanamycin. This entry clone was subsequently isolated and recombined with the pASGW-attR destination vector in an LR clonase reaction to form the final pASGW::tW53 expression clone, which was transformed into competent DH5α cells and selected for on LB media containing 100 μg mL^−1^ ampicillin. The expression clone was sequenced to verify the integrity of the reading frame and tested for autoactivation and cytotoxicity by transforming into Y2HGold yeast cells using the Frozen-EZ Yeast Transformation II kit (Zymo Research, Orange, CA, USA), and plating onto SD/–Trp single dropout media supplemented with either 20 ng mL^−1^ X-α-gal (Gold Biotechnology, St. Louis, MO, USA) or X-α-gal and 125 ng mL^−1^ Aureobasidin A (Clontech). After co-transformation of 1 μg each of bait and prey vector into Y2HGold yeast cells, the cells were grown on SD/–Leu/–Trp double dropout media supplemented with X-α-gal (DDOX). Blue colonies were selected and replica plated onto SD/–Ade/–His/–Leu/–Trp quadruple dropout media supplemented with X-α-gal and Aureobasidin A (AurA) (QDOXA). This selects for the presence of BD vector (–Trp), AD vector (–Leu) and the activation of the four reporter genes *HIS3* (–His), *ADE2* (–Ade), *MEL1* (X-α-gal) and *AUR1-C* (AurA). AD vector plasmids were rescued from yeast clones showing positive interactions by scraping colonies from plates into 67 mM of KH_2_PO_4_ and digesting with 30 U of zymolase (Seikagaku, Tokyo, Japan) for 1 h at 37 °C. Digestion was followed by column purification with a QIAprep Spin Miniprep kit (Qiagen, Hilden, Germany). Isolated plasmids were subcloned into DH5α *E. coli* in order to obtain a higher yield and tested for the presence of cDNA inserts by PCR with AD_fwd and AD_rvs primers (Table 4) before being sequenced.

### Identification of potential *WRKY53* targets

Potential target promoters for the TaWRKY53 transcription factor were identified by mining the rice genome for genes with putative functional linkages to LOC_Os05g27730 (*OsWRKY53*) via the RiceNet probabilistic functional gene network (Lee et al. 2011). The Gene Coexpression Analysis tool from the MSU Rice Genome Annotation Project Database (Childs et al. 2011) was used to identify genes with expression profiles correlated to that of *OsWRKY53* during an infection time course with the hemibiotrophic fungal pathogen *M. oryzae* (Marcel et al. 2010). Gene Ontology (GO) SLIM assignments for all predicted rice genes were obtained from the MSU Rice Genome Annotation Project (http://rice.plantbiology.msu.edu/downloads.shtml). GO term enrichment tests were performed using a modified Fisher’s exact test, and modified EASE scores were calculated to indicate significant enrichment of GO terms in the coregulated genes (*n* = 96) compared to the genome background (*n* = 67,393) (Hosack et al. 2003; Huang et al. 2008). The 1 kb upstream promoter regions of all predicted gene models in the MSU Rice Genome Annotation Project v6.1 were screened for the presence of the W-box (C/T)TGAC(C/T) consensus motif using a custom Perl script, and numbers of W-box motifs per gene model were calculated. Frequency distributions of numbers of W-box motifs per gene model were calculated and compared between the coregulated gene subset and the genome-wide background with a Mann-Whitney-Wilcoxon test (*p* value = 3.288e-13).

### TaWRKY53 protein expression

The wheat WRKY53 protein was expressed with the Champion pET SUMO protein expression system (Invitrogen) for use in electrophoretic mobility shift assays (Panavas et al. 2009). The *TaWRKY53* CDS was amplified with primers TaWRKY53exp_fwd1 and TaWRKY53_exp_rvs1 (Table 1) from a cloned full-length cDNA template previously isolated from GR wheat. The purified amplification product was ligated to the pET Sumo vector and transformed into Mach1-T1R chemically competent *E. coli*. Once the recombinant plasmid pET::W53 was isolated and sequenced to verify the N-terminal in-frame fusion of the *TaWRKY53* CDS with the SUMO tag, the plasmid was transformed into competent BL21(DE3) *E. coli* for expression. Fresh LB medium containing 50 μg mL^−1^ kanamycin and 1 % glucose was inoculated at a ratio of 1:50 with overnight culture and grown at 37 °C until mid-log phase (OD600 = 0.5). Protein expression was induced with 1 mM IPTG and the culture incubated for a further 4.5 h before bacterial cell lysates were prepared. SUMO::TaWRKY53 fusion protein was purified using the N-terminal polyhistidine (6× His) tag and ProBond Ni^2+^-chelating resin (Invitrogen), following the manufacturer’s hybrid purification protocol to ensure maximum solubility and biological activity. TaWRKY53 encompasses 439 amino acids and is calculated to be a 47.39-kDa protein. Therefore, the SUMO::TaWRKY53 fusion protein is expected to be ~60 kDa in size. Protein yield was determined via a Pierce 660 nm protein assay (Thermo Scientific, Rockford, IL, USA) and was visualized using 10 % polyacrylamide gel electrophoresis. Protein concentration was adjusted to 500 μg mL^−1^ in 30 % glycerol.

### Electrophoretic mobility shift assay

Three rice genes from different functional categories, each with four or more W-boxes, were selected for in vitro binding assays with the expressed TaWRKY53 protein. Based on the 1 kb upstream sequence information of these genes, biotinylated double-stranded DNA probes 80 bp in length were synthesized (Integrated DNA Technologies, Coralville, IA, USA) (Table 3). Binding assays were performed using the LightShift Chemiluminescent electrophoretic mobility shift assays (EMSA) kit (Thermo Scientific, Barrington, IL, USA). A total of 500 μg of purified TaWRKY53 protein was incubated with each probe in binding buffer (10 mM Tris, 50 mM KCl, 1 mM dithiotreitol, pH 7.5) and incubated for 20 min at room temperature. Protein was either incubated with 0.08 ng of labelled probe alone, or with labelled probe and 200 ng of unlabelled probe. Binding reactions were separated on 6 % acrylamide/0.5× TBE non-denaturing gels, transferred to nylon membranes and blocked, washed and detected according to the manufacturer’s instructions. Membranes were placed in a film cassette and exposed to X-ray film for 5 min. An alternative EMSA was also performed (Fig. S3) by using primers (Table S3) to amplify larger, 1-kb rice promoter fragments from the genomic DNA of *O. sativa* ssp. japonica cv. ‘Nipponbare’. Approximately 1 μg of each promoter fragment was combined with 500 μg of protein and incubated in binding buffer (10 mM Tris, 100 mM KCl, 1 mM EDTA, 0.1 mM DTT, 5 % *v*/*v* glycerol, 0.01 mg mL^−1^ BSA, pH 7.5) at room temperature for 20 min (Hellman and Fried 2007). The reactions were separated on 1 % agarose gel in TAE buffer (Berman et al. 1987) and stained using SYBR Green I nucleic acid gel stain (Invitrogen) according to the manufacturer’s instructions.

## Results

### *WRKY53* sequence features are remarkably well-conserved

**Fig. 1:**
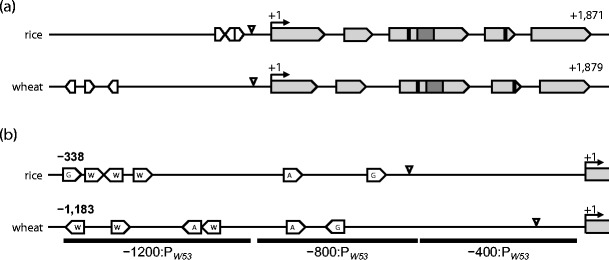
**a** Gene models for the *WRKY53* orthologs in rice and wheat: both orthologs span five exons, represented by *grey arrows*; the two conserved WRKY domains are indicated by *black bars*; the zinc finger motif is indicated by a *dark grey bar*. The relative promoter positions of the W-box WRKY transcription factor binding motifs are indicated by *open arrows*. **b** Features of the promoter regions of the rice and wheat orthologs of *WRKY53*. Putative *cis*-acting regulatory elements are indicated by *open arrows*: *A* ABRE abscisic acid-responsive element; *G* GCC-box ethylene-responsive element; *W* W-box WRKY transcription factor binding motif. The regions amplified from *TaWRKY53* for use in yeast one-hybrid assays are indicated by *horizontal black bars*

We amplified the entire 1,879 nucleotide open reading frame and 1,211 nucleotides of upstream promoter sequence for the wheat ortholog of *WRKY53* (GenBank accession KC174859). Our primers (Table 1) did not detect multiple transcripts, and LOC_Os05g27730, encoding rice ortholog *OsWRKY53*, is predicted to produce only a single splicing variant. Aligning the wheat genomic and cDNA sequences allowed for the mapping of intron-exon boundaries and the construction of a gene model for *TaWRKY53,* which was then compared to the gene model for *OsWRKY53* (Fig. 1a). The coding region of *TaWRKY53* spans five exons, similar to the intron-exon pattern predicted for *OsWRKY53*. Exons 3 and 4 encode the two conserved WRKY domains, consistent with its classification as a group Ia WRKY protein (Zhu et al. 2013); exon 3 also includes a zinc finger motif, which conforms to the CX_[4–5]_CX_[22–23]_HXH consensus sequence for Cys_2_–His_2_-type zinc fingers; the last three codons of exon 3 and the first codon of exon 4 encode the nuclear localization signal. Similar to *AtWRKY33*, the ortholog from *Arabidopsis thaliana* (Mao et al. 2011), there are several putative phosphorylation sites in the N-terminal region (Fig. S1).

The 1.2-kb upstream promoter sequences of *TaWRKY53* and *OsWRKY53* were inspected for the presence of *cis*-acting regulatory motifs. Although the motifs found are similar between wheat and rice, their number, orientation and distance from the start of translation varies (Fig. 1b). Two abscisic acid-responsive elements (ABRE) present in the *TaWRKY53* promoter conform to the (A/C)ACG(C/T)GC motif consensus, at −655 and −875 bp, and a GCC-box ethylene-responsive element conforms to the AGCCGCC motif, at −567 bp upstream. By contrast, the rice promoter has only one ABRE motif, at −189 bp, but two GCC-boxes at −135 and −332 upstream. The *TaWRKY53* promoter contains three W-box elements that conform to the (C/T)TGAC(C/T) consensus motif, at −869, −1,064 and −1,178 bp upstream of the ATG translation initiation codon (Fig. 1b). The *OsWRKY53* promoter has a similar number of W-boxes, at −298, −316 and −322 bp (Chujo et al. 2009).

### Proteins interacting with the *WRKY53* promoter and the WRKY53 protein

**Fig. 2:**
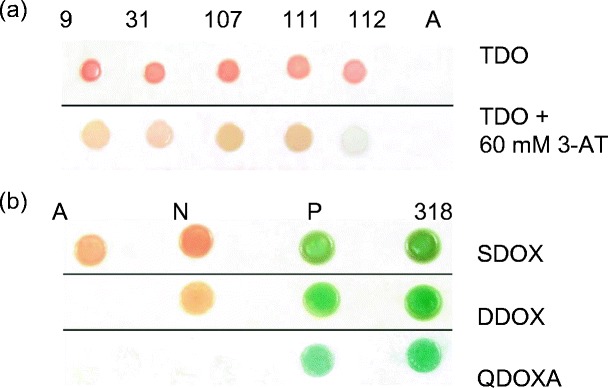
**a** Yeast one-hybrid interactions. Colonies 9–111 have the *HIS3* reporter gene under the control of the −400:P_*W53*_ promoter segment. Colony 112 harbours an empty prey protein vector and acts as a negative control. The identities of the interactors are summarized in Table 2. *A* autoactivation control, *TDO* triple dropout SD/–His/–Leu/–Ura media, *3-AT* 3-amino–1,2,4-triazole. **b** Yeast two-hybrid interactions. All colonies express the truncated WRKY53 protein from the pASGW::tW53 vector. *Blue colour* indicates the activation of the *MEL1* gene. *A* autoactivation control, *N* negative control, *P* positive control; 318, positive interactor, a microsomal glutathione *S*-transferase. *SDOX* single dropout SD/–Trp/X-α-gal media; *DDOX* double dropout SD/–Leu/–Trp/X-*α*-gal media; *QDOXA* quadruple dropout SD/–Leu/–Trp/–Ade/–His/X-*α*-gal/AurA media

To discover possible transcriptional regulators for *WRKY53*, and to test whether such interactions are conserved across cereals, constructs were made for yeast one-hybrid assays with three discrete segments of the 1.2-kb promoter region of *TaWRKY53*, −400:P_*W53*_, −800:P_*W53*_ and −1200:P_*W53*_ (Fig. 1b). These were used as DNA bait against a previously constructed rice pathogen-responsive cDNA expression library (Niño-Liu et al. 2005). A total of four positive interactors with −400:P_*W53*_ were identified (Fig. 2a): LOC_Os01g72100, encoding *OsCML10*; LOC_Os08g42850, encoding *OsFKBP16-3*; LOC_Os07g47640, encoding an ultraviolet B-repressible protein; and LOC_Os04g45834, encoding a DUF584 domain-containing protein. Because of autoactivation of the −800:P_*W53*_ and −1200:P_*W53*_ DNA bait strains, likely by endogenous yeast proteins, these promoter segments were excluded from our analysis. Sequencing results for the four *WRKY53* promoter interactors are summarized in Table 2. We also investigated protein–protein interactions with WRKY53 to identify additional components of its transcriptional network. We expressed a truncated TaWRKY53 protein lacking the first 180 amino acids to prevent autoactivation (Lai et al. 2011). This truncated wheat protein was fused to the GAL4 binding domain for yeast two-hybrid assays against the same rice prey vector library used for yeast one-hybrid analysis. Of more than 200 individual clones obtained, only one clone, 318, maintained strong growth and reporter gene activation upon repeated replica plating on high stringency media (Fig. 2b). We speculate that this high level of stringency (requiring the activation of four separate reporter genes) could explain why only a single clone was obtained. However, a bait protein from wheat may bind to rice proteins less efficiently than a rice homolog, and the truncation of that wheat bait protein may also have negatively affected the ability of our assay to discover proteins interacting with WRKY53. The cDNA expressed by 318 had significant homology to LOC_Os03g50130, which encodes a putative microsomal glutathione *S*-transferase 3 (Table 2).

**Table 2:** Yeast-hybrid interactors

Interaction	Bait	Clone ID	Homology^a^	E-value
One-hybrid	−400:P_*W53*_	9	LOC_Os01g72100 *OsCML10* calmodulin-related calcium sensor protein	3.9e^−59^
		31	LOC_Os08g42850 FKBP-type peptidyl-prolyl *cis-trans* isomerase	3.9e^−104^
		107	LOC_Os07g47640 ultraviolet B-repressible protein	1.3e^−132^
		111	LOC_Os04g45834 DUF584 domain-containing protein	1.1e^−10^
Two-hybrid	tW53	318	LOC_Os03g50130 microsomal glutathione *S*-transferase 3	3.9e^−59^

^a^Homology based on BLASTn searches of the MSU Rice Genome Annotation Project database

### Potential target genes from *WRKY53* coexpression networks

Following the rationale that genes that function together have similar expression profiles, the RiceNet Probabilistic Functional Gene Network (Lee et al. 2011) and MSU Gene Coexpression Analysis (Childs et al. 2011) tools were used to identify candidate genes potentially regulated by *WRKY53.* RiceNet returned 36 loci linked to LOC_Os05g27730 which encodes *OsWRKY53* (Fig. S2a), with coherence scores ranging from 1.11 (LOC_Os03g01740) to 3.74 (LOC_Os04g34140). The MSU Gene Coexpression Analysis tool indicated that a total of 62 loci out of 1,161 in the *M. oryzae*-induced dataset were correlated with *OsWRKY53* expression at a very stringent cut-off of between 0.99 and 1 (Fig. S2b). Interestingly, only four loci (LOC_Os09g37080, LOC_Os03g53020, LOC_Os03g01740 and LOC_Os01g38980) were present in both analyses. Two defence-related genes upregulated in *OsWRKY53*-overexpressing transgenic rice cells (Chujo et al. 2007) were also included to generate a combined set of 96 potential targets for *WRKY53* (Table S1).

**Fig. 3:**
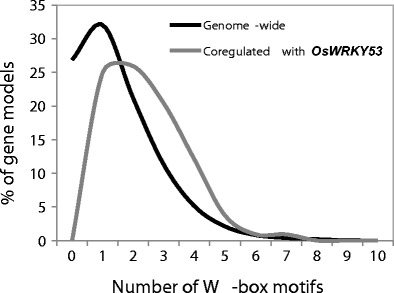
Frequency distributions of numbers of W-box motifs for all predicted gene models in the MSU rice genome annotation v6.1 (*n* = 67,393) compared to a subset of genes coregulated with *OsWRKY53* (*n* = 96)

The 1-kb promoters of these *WRKY53*-coregulated loci are enriched for the presence of W-box WRKY binding motifs when compared to the frequency distribution of all gene models across the rice genome (Fig. 3). This provides further evidence that some of these coregulated genes may be downstream targets of *WRKY53*. The list of coregulated loci exhibits a significant enrichment for Gene Ontology (GO) terms corresponding to molecular functions such as kinase activity, DNA binding and transcription regulator activity, and biological processes such as response to stress, response to extracellular stimulus, and signal transduction (Table S2), consistent with the function of *WRKY53* as a transcription factor involved in stress responses.

**Fig. 4:**
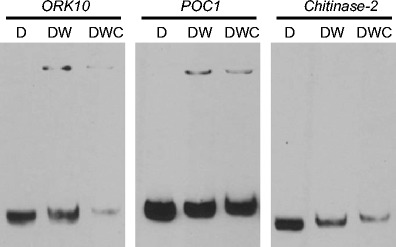
Electrophoretic mobility shift assays. *D* biotinylated DNA fragment, *W* expressed TaWRKY53 protein, *C* unlabeled competitor DNA

To address whether the promoters of bioinformatically determined target genes can be bound by the WRKY53 protein, and also whether binding specificity is conserved across cereals, we designed DNA probes based on promoter sequences of three coregulated rice genes (Tables 3 and 4). These were used as targets for binding in electrophoretic mobility shift assays (EMSAs) with an expressed wheat WRKY53 protein (Fig. 4). All three genes selected have distinct functions in plant disease resistance: *chitinase-2* (LOC_Os11g47600), a PR (pathogenesis-related) protein expressed in response to biotic stressors such as aphids and pathogenic fungi (Van der Westhuizen et al. 1998; Akimoto-Tomiyama et al. 2003; Ramonell et al. 2005; Chujo et al. 2007); the Ser/Thr-type receptor kinase *ORK10* (LOC_Os01g02300), induced in cereals infected by biotrophic rust fungi (Feuillet et al. 1997; Cheng et al. 2002; Marcel et al. 2010); and the apoplastic cationic peroxidase *POC1* (LOC_Os07g48050), induced by *X. oryzae* pv. *oryzae* and aphid infestation as part of the oxidative burst (Young et al. 1995; Van der Westhuizen et al. 1998; Hilaire et al. 2001; Anguelova-Merhar et al. 2002). Binding of the wheat WRKY53 to the promoter fragments of *ORK10* and *POC1* was observed, but not to the promoter fragment of *chitinase-2*. An alternative EMSA using longer, 1-kb promoter fragments also did not demonstrate binding of WRKY53 to *chitinase-2* (Fig. S3), similar to the lack of binding observed with *POX5.1* (LOC_Os07g48040) (Chittoor et al. 1997), a peroxidase related to *POC1* but with no W-boxes in its 1-kb promoter. This might indicate the requirement for additional cofactors or phosphorylation of WRKY53 for binding to the W-boxes in the *chitinase-2* promoter to occur (Wan et al. 2004; Mao et al. 2011). However, the interaction between wheat WRKY53 and the rice *ORK10* and *POC1* promoters in our assay demonstrates that putative genetic interactions determined through bioinformatic analysis can be substantiated by simple in vitro DNA–protein binding assays and that such binding predictions can be translated across related cereals.

**Table 3:** Electrophoretic mobility shift assay probes

Locus ID	Annotation	EMSA fragment^a^
LOC_Os11g47600	*chitinase 2*	AGCCTCACGTTTCGTCCTGATTGCAAGTTG**TTGACT**TAAAT**TTGACT**TGTCTCGGAACAAAACAATAACCTGCAGTCCGT
LOC_Os01g02300	*ORK10* kinase	ATCT**GGTCAA**CAATGTATTACACACTGCT**TTGACT**ACTTCCCCCAAAAAAGTACACACTGCT**TTGACT**CA**GGTCAA**ACTT
LOC_Os07g48050	*POC1* peroxidase	ACGTAAATTTTTTGAATAAGACAAAT**GGTCAA**ACATGTAAGAAAAGAA**AGTCAA**CGGCGTCATCTATTTAAAAAACGGAT

^a^The presence of W-boxes is indicated in bold

**Table 4:** Primers used for the construction of yeast-hybrid vectors

Primer ID	Primer sequence
attB1-tW53_fwd	GGGGACAAGTTTGTACAAAAAAGCAGGCTTATACAATTGGAGGAAGTACGGGCAG
attB2-W53_rvs	GGGGACCACTTTGTACAAGAAAGCTGGGTCCTACTAGCAGAGGAGCGACTCGACGAA
attB4-Pw53–400_fwd	GGGGACAACTTTGTATAGAAAAGTTGTCTCGATTGATTGCCCGCACCAAA
attB1R-Pw53-400_rvs	GGGGACTGCTTTTTTGTACAAACTTGACCGACGGTACATGCCATAGGTCC
attB4-Pw53-800_fwd	GGGGACAACTTTGTATAGAAAAGTTGCGTGTTGGTGCAGCCATCTCGTAT
attB1R-ppw53-800_rvs	GGGGACTGCTTTTTTGTACAAACTTGTGCGGGGTTTGTTTTACTCTGGAA
attB4-Pw53-1200_fwd	GGGGACAACTTTGTATAGAAAAGTTGATCAGGGTCTGGCGTAGTCAGGTG
attB1R-Pw53-1200_rvs	GGGGACTGCTTTTTTGTACAAACTTGGCATGGTACATCCCCGACCTGAGA
AD_fwd	CTATTCGATGATGAAGATACC
AD_rvs	GTGAACTTGCGGGGTTTTTCA

## Discussion

The presence of abscisic acid-responsive elements (ABRE) and GCC-box ethylene-responsive elements in the promoter regions of *TaWRKY53* and *OsWRKY53* (Fig. 1b) supports the function of the *WRKY53* transcription factor in stress regulation, since abscisic acid-responsive genes are upregulated during drought (Christmann et al. 2006) and aphid infestation (Park et al. 2006), and GCC-box elements are a hallmark of the promoters of aphid- and pathogen-responsive genes (Rushton and Somssich 1998; Smith and Boyko 2007; Dong et al. 2010). We speculate that similar sets of regulatory factors will be recruited to the promoters of *TaWRKY53* and *OsWRKY53*, although the precise number and position of *cis*-acting elements might impact binding efficiency and contribute to any presumed interspecific differences between these orthologs. The presence of W-boxes in the *TaWRKY53* and *OsWRKY53* promoters implies either regulation by other WRKY transcription factors (Rushton et al. 2010; Eulgem and Somssich 2007), or autoregulation as has been described for the orthologs *AtWRKY33* in *Arabidopsis* (Mao et al. 2011) and *PcWRKY1* in parsley (*Petroselinum crispum* L.) (Turck et al. 2004). These W-boxes are required for elicitor responsiveness of *OsWRKY53* (Chujo et al. 2009) and pathogen-responsive induction of *AtWRKY33* (Lippok et al. 2007). Although the W-boxes are located much further upstream in *TaWRKY53*, their overall number and orientation resembles that found in orthologs from other plant species (Turck et al. 2004; Lippok et al. 2007). This extensive interspecies preservation of gene architecture and specific regulatory elements is in agreement with phylogenetic evidence (Wu et al. 2008; Zhu et al. 2013) and suggests strong conservation of function and a pivotal role in plant stress responses. It is therefore likely that the rice and wheat orthologs of *WRKY53* target equivalent sets of stress response genes.

**Fig. 5:**
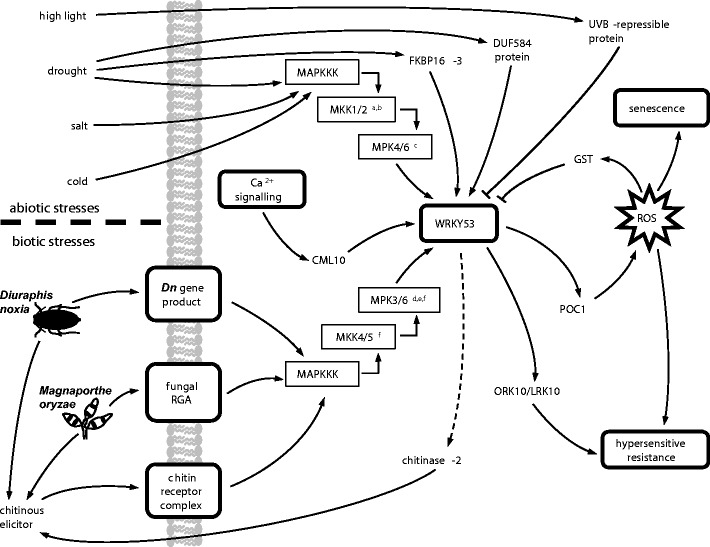
A gene network for *WRKY53* in cereals. The *WRKY53* transcription factor is able to receive many types of stress inputs, from both abiotic and biotic pathways, and transduce those to an appropriate oxidative burst. Our study suggests that *CML10*, *FKBP16-3*, a *DUF584 protein* and a *UVB-repressible protein* are upstream regulatory components, whereas *GST*, *ORK10/LRK10* and the peroxidase *POC1* are downstream targets of *WRKY53* regulation. ^a^Ichimura et al. (1998), ^b^Teige et al. (2004), ^c^Qiu et al. (2008), ^d^Wan et al. (2004), ^e^Mao et al. (2011), ^f^Asai et al. (2002)

All four proteins that interacted with the *WRKY53* promoter in the yeast one-hybrid assays (Table 2) are known to be involved in plant stress responses: *OsFKBP16-3* (LOC_Os08g42850), an FKBP-type peptidyl-prolyl *cis-trans* isomerase (PPI) that functions in osmotic stress tolerance (Ahn et al. 2010; Gollan and Bhave 2010); *OsCML10* (LOC_Os01g72100), a member of a calmodulin-related calcium sensor protein family known to interact with transcription factors (Popescu et al. 2007; Reddy and Reddy 2004); an ultraviolet B-repressible protein (LOC_Os07g47640) that serves as a *trans*-acting negative regulator of stress-responsive genes (Olbrich et al. 2005); and a protein containing a DUF584 domain (LOC_Os04g45834) found in several DNA-binding senescence-related proteins (Fischer-Kilbienski et al. 2010; Krupinska et al. 2002). We therefore conclude that *WRKY53* can receive inputs from several stress-related pathways (Fig. 5), including those affording responsiveness to calcium signalling mechanisms initiated by pest and pathogen detection, or to environmental cues such as drought stress and ultraviolet radiation. This is supported by our analyses demonstrating interactions between the *WRKY53* promoter and OsCML10, OsFKBP16-3, and an ultraviolet-repressible protein. Evidence from the literature that *WRKY53* is highly induced in senescing wheat leaves (Wu et al. 2008) is in accord with our discovery of a protein containing the senescence-related DUF584 domain as a regulator of WRKY53 expression. Additionally, all four of these interactors belong to gene classes that are coregulated with *WRKY* genes during various abiotic and biotic stress responses (Izaguirre et al. 2003; Wang et al. 2007; Galon et al. 2008; Qiu et al. 2008; Fischer-Kilbienski et al. 2010).

There is also mounting evidence that glutathione *S*-transferases (GSTs) form an integral part of WRKY transcriptional networks (Hahn and Strittmatter 1994; Olbrich et al. 2005; Shimono et al. 2007; Encinas-Villarejo et al. 2009). This supports our yeast two-hybrid data which indicates that a microsomal glutathione *S*-transferase 3 is able to interact with the WRKY53 protein (Table 2). GSTs play a role in ameliorating oxidative damage (Jakobsson et al. 1999; Gill and Tuteja 2010) and are responsible for scavenging free radicals in the wake of the SA-mediated oxidative burst in response to abiotic stress (Cummins et al. 2011) and pathogen and aphid attack (Lieberherr et al. 2003; Couldridge et al. 2007; Botha et al. 2005; Moloi and van der Westhuizen 2008). Considering that *D. noxia* feeding induces chlorosis and oxidative damage to cereal leaves (Ni et al. 2001; Ni and Quisenberry 2003) and that *TaWRKY53* is essential for aphid resistance in wheat (Van Eck et al. 2010), our yeast two-hybrid data provide evidence that membrane-bound glutathione *S*-transferases might alter the reactive oxygen species (ROS) response in a *TaWRKY53*-mediated way. This could be achieved either through the induction of detoxifying gene products to protect the photosynthetic machinery from free-radical damage, or by quenching runaway ROS production during the hypersensitive response.

The *Arabidopsis* ortholog *AtWRKY33* is phosphorylated by the stress-responsive mitogen-activated protein kinases (MAPKs) MPK3 and MPK6 (Wan et al. 2004; Mao et al. 2011). Although orthologs of these MAPKs were not detected in our yeast two-hybrid assay, their inclusion in the working model (Fig. 5) of the WRKY53 transcriptional network is warranted; because of the evolutionary conservation of MAPK modules even between distantly related species (Asai et al. 2002; Hamel et al. 2006), *WRKY53* remains a plausible target of such kinase signalling cascades in cereals, with its activity perhaps modulated by stress-responsive interactors such as the GST we identified.

We infer from our data that *WRKY53* is able to transduce this wide range of stress-responsive signals to several downstream targets. Particularly prominent in our study is the involvement of the oxidative burst and genes forming part of the pathogen defence repertoire, including *GST*, *ORK10* and *POC1* (Fig. 4)*. ORK10/LRK10* is a receptor kinase important in the fungal resistance response of cereals (Feuillet et al. 1997; Cheng et al. 2002; Marcel et al. 2010), and *POC1* is an apoplastic peroxidase induced as part of the oxidative burst in response to aphids or pathogens (Young et al. 1995; Van der Westhuizen et al. 1998; Hilaire et al. 2001; Anguelova-Merhar et al. 2002). This is consistent with a regulatory role for *OsWRKY53* during rice blast infection (Chujo et al. 2007) and *TaWRKY53* during the resistance response to aphid attack (Van Eck et al. 2010) and implies that *WRKY53* is a regulator of ROS release during the hypersensitive response (Fig. 5).

In summary, we have demonstrated that the gene structure and *cis*-acting regulatory elements of *WRKY53* are highly conserved between wheat and rice, and report several novel genes that act as either upstream regulators affording *WRKY53* responsiveness to various biotic and abiotic stress inputs, or downstream targets involved in oxidative responses to stress.

## Electronic supplementary material

Below is the link to the electronic supplementary material.

Fig. S1 Translated peptide sequence from a full-length *TaWRKY53* cDNA clone isolated from hexaploid wheat (*Triticum aestivum* L.) cv. ‘Gamtoos-R’. The protein consists of 439 amino acid residues. The five putative N-terminal phosphorylation sites are highlighted in bold, the two conserved WRKY domains are highlighted in bold, the zinc-finger motif is underlined and the nuclear localization signal is boxed. (PDF 28 kb)

Fig. S2 *WRKY53* coexpression networks determined *in silico*. (a) RiceNet CytoScape probabilistic functional gene network indicating the presence of 36 loci coexpressed with LOC_Os05g27730, encoding *OsWRKY53*. (b) MSU Rice Genome Annotation Project Gene Coexpression Analysis. A total of 62 loci out of 1,161 in the *M. oryzae* infection-induced dataset were coregulated with LOC_Os05g27730 at a correlation >0.99, indicated in red. (PDF 98 kb)

Fig. S3 SYBR Green I-based electrophoretic mobility shift assay using large 1 kb promoter fragments and expressed TaWRKY53 protein. The addition of TaWRKY53 protein is indicated by (+) and lanes with only DNA are indicated by (–). (PDF 37 kb)

Table S1 Rice loci coregulated with *OsWRKY53*. (PDF 39 kb)

Table S2 Gene ontology (GO) enrichment test results comparing a subset of rice genes coregulated with LOC_Os05g27730 to the genome-wide background. A, B and C, GO term ID, name, and category; D, number of genes with the GO term in gene subset; E, number of genes with the GO term in background gene set; F and G, numbers of genes without GO term in subset and background; H, *p*-value of enrichment test: *p*-values of significantly enriched GO terms are in bold; I, list of genes with the GO term in the gene subset. (XLSX 13 kb)

Table S3 Primers used for SYBR Green I-based electrophoretic mobility shift assay. (PDF 23 kb)
