# Supplementary material for: The transcriptional network of WRKY53 in cereals links oxidative responses to biotic and abiotic stress inputs
Source: Funct Integr Genomics. 2014 Apr 29;14(2):351–62. doi: 10.1007/s10142-014-0374-3 (PMC4059961; doi:10.1007/s10142-014-0374-3)
Supplement: Supplementary file 1 — Translated peptide sequence from a full-length TaWRKY53 cDNA clone isolated from hexaploid wheat (Triticum aestivum L.) cv. ‘Gamtoos-R’. The protein consists of 439 amino acid residues. The five putative N-terminal phosphorylation sites are highlighted in bold, the two conserved WRKY domains are highlighted in bold, the zinc-finger motif is underlined and the nuclear localization signal is boxed. (PDF 28 kb) [file 10142_2014_374_MOESM1_ESM.pdf]

SUPPORTING INFORMATION

1 MSSSTGSLDHAGFTFTPPPFITSFTELLSGSGAGDAER**S**PRGFN

45 RGGRAGAPKFKSAQPPSLPIS**S**PFSCFSVPAGL**S**PAELLD**S**PVL

89 LNYSHILA**S**PTTGAIPAQRCDWQASADLNTFQQDELGLSGFSFH

133 AVKSNATVNAQANRLPLFKEQQEQQQEEVVQVSNKSSSSGNNKQ

177 VEDGYN**WRKYGQK**QVKGSENPRSYYK<u>CTYNNCSMKKKVERSLAD</u>

221 <u>GRITQIVYKGAHDH</u>PKPLSTRRNSSGCAAVVAEDHTNGSEHSGP

265 TPENSSVTFGDDEADKPETKRRKEHGDNEGSSGGTGGCGKPVRE

309 PRLVVQTLSDIDILDDGFR**WRKYGQK**VVKGNPNPRSYYKCTTVG

353 CPVRKHVERASHDNRAVIATYEGKHSHDVPIGRGRALPASSSSD

397 SSAVIWPAAAVQAPCTLEMLAGHPGYAAKDEPRDDMFVESLLC*

Figure S1. Translated peptide sequence from a full-length *TaWRKY53* cDNA clone isolated from hexaploid wheat (*Triticum aestivum* L.) cv. ‘Gamtoos-R’. The protein consists of 439 amino acid residues. The five putative N-terminal phosphorylation sites are highlighted in bold, the two conserved WRKY domains are highlighted in bold, the zinc-finger motif is underlined and the nuclear localization signal is boxed.
