# Supplementary material for: The transcriptional network of WRKY53 in cereals links oxidative responses to biotic and abiotic stress inputs
Source: Funct Integr Genomics. 2014 Apr 29;14(2):351–62. doi: 10.1007/s10142-014-0374-3 (PMC4059961; doi:10.1007/s10142-014-0374-3)
Supplement: Supplementary file 3 — SYBR Green I-based electrophoretic mobility shift assay using large 1 kb promoter fragments and expressed TaWRKY53 protein. The addition of TaWRKY53 protein is indicated by (+) and lanes with only DNA are indicated by (–). (PDF 37 kb) [file 10142_2014_374_MOESM3_ESM.pdf]

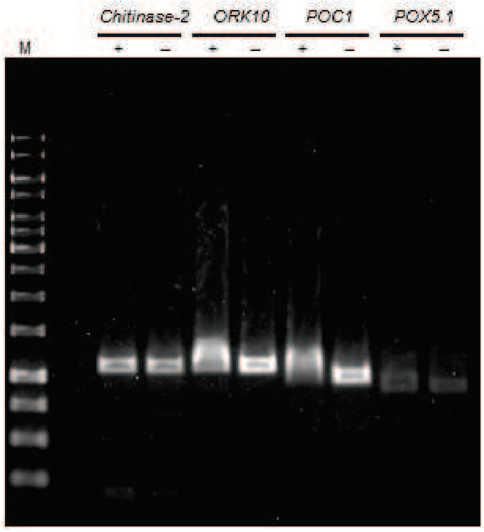

Figure S3. SYBR Green I-based electrophoretic mobility shift assay using large 1 kb promoter fragments and expressed TaWRKY53 protein. The addition of TaWRKY53 protein is indicated by (+), and lanes with only DNA are indicated by (–).
