# Supplementary material for: The transcriptional network of WRKY53 in cereals links oxidative responses to biotic and abiotic stress inputs
Source: Funct Integr Genomics. 2014 Apr 29;14(2):351–62. doi: 10.1007/s10142-014-0374-3 (PMC4059961; doi:10.1007/s10142-014-0374-3)
Supplement: Supplementary file 4 — Rice loci coregulated with OsWRKY53. (PDF 39 kb) [file 10142_2014_374_MOESM4_ESM.pdf]

Table S1. Rice loci coregulated with *OsWRKY53*

Locus ID	W-Boxes	Functional Annotation
LOC_Os01g61080	8	OsWRKY24
LOC_Os03g47280	6	VQ domain containing protein, putative, expressed
LOC_Os01g02300	5	receptor kinase ORK10, putative, expressed
LOC_Os01g27590	5	transposon protein, putative, Pong sub-class, expressed
LOC_Os03g58010	5	acetyltransferase
LOC_Os05g50180	5	OsCML14 - Calmodulin-related calcium sensor protein, expressed
LOC_Os01g03690	4	TKL_IRAK_DUF26-lg.1 - DUF26 kinases have homology to DUF26 containing loci, expressed
LOC_Os02g09960	4	Lyk8
LOC_Os03g37090	4	expressed protein
LOC_Os03g52410	4	expressed protein
LOC_Os04g46240	4	AP2 domain prot
LOC_Os05g41780	4	AP2 domain prot
LOC_Os05g46840	4	proline-rich protein, putative, expressed
LOC_Os06g13180	4	metalloendoproteinase 1 precursor, putative, expressed
LOC_Os07g48050	4	peroxidase precursor, putative, expressed
LOC_Os08g04370	4	plastocyanin-like domain containing protein, putative, expressed
LOC_Os11g36200	4	receptor-like protein kinase 2 precursor, putative, expressed
LOC_Os11g47600	4	glycosyl hydrolase (chitinase)
LOC_Os12g41110	4	OsCML5 - Calmodulin-related calcium sensor protein, expressed
LOC_Os01g06280	3	TKL_IRAK_CrRLK1L-1.4 - The CrRLK1L-1 subfamily has homology to the CrRLK1L homolog, expressed
LOC_Os01g46800	3	OsWRKY15 - Superfamily of TFs having WRKY and zinc finger domains, expressed
LOC_Os01g56240	3	OsSAUR2 - Auxin-responsive SAUR gene family member, expressed
LOC_Os01g67810	3	transposon protein, putative, unclassified, expressed
LOC_Os01g74250	3	TIGR01615 protein
LOC_Os02g02600	3	serine/threonine-protein kinase Cx32, chloroplast precursor, expressed
LOC_Os02g03410	3	CAMK_CAMK_like.12 - CAMK includes calcium/calmodulin depedent protein kinases, expressed

LOC_Os02g11859	3	expressed protein
LOC_Os02g13220	3	F-box family protein, putative, expressed
LOC_Os02g15810	3	HMG1/2
LOC_Os02g22160	3	DNA binding protein, putative, expressed
LOC_Os02g37330	3	heavy metal associated domain containing protein, expressed
LOC_Os02g45780	3	C3HC4 zinc finger
LOC_Os03g02514	3	hydrolase, alpha/beta fold family protein, putative, expressed
LOC_Os03g15770	3	tyrosine protein kinase domain containing protein, putative, expressed
LOC_Os05g01940	3	zinc finger, RING-type, putative, expressed
LOC_Os05g39930	3	spotted leaf 11, putative, expressed
LOC_Os06g04230	3	expressed protein
LOC_Os07g10970	3	leucine zipper protein-like, putative, expressed
LOC_Os07g39720	3	expressed protein
LOC_Os07g48010	3	peroxidase precursor, putative, expressed
LOC_Os09g37080	3	expressed protein
LOC_Os01g28790	2	PRAS-rich protein, putative, expressed
LOC_Os01g38980	2	calmodulin-binding protein, putative, expressed
LOC_Os02g33590	2	U-box domain-containing protein, putative, expressed
LOC_Os02g33680	2	U-box domain containing protein, expressed
LOC_Os02g50490	2	endoglucanase, putative, expressed
LOC_Os02g54600	2	STE_MEK_ste7_MAP2K.5 - STE kinases
LOC_Os03g53020	2	helix-loop-helix DNA-binding domain containing protein, expressed
LOC_Os03g58020	2	acetyltransferase, GNAT family, putative, expressed
LOC_Os04g03920	2	expressed protein
LOC_Os04g33390	2	prephenate dehydratase domain containing protein, expressed
LOC_Os04g34030	2	U-box domain-containing protein, putative
LOC_Os04g34050	2	VQ domain containing protein, putative, expressed
LOC_Os05g03620	2	TKL_IRAK_CR4L.4 - The CR4L subfamily has homology with Crinkly4, expressed
LOC_Os05g08830	2	expressed protein
LOC_Os05g45410	2	HSF-type DNA-binding domain containing protein, expressed

Os06g14450	2	exo70 exocyst complex subunit family protein, putative, expressed
Os06g44010	2	OsWRKY28
Os07g34940	2	aspartic proteinase nepenthesin-1 precursor, putative, expressed
Os07g35280	2	TKL_IRAK_DUF26-lc.1 - DUF26 kinases have homology to DUF26 containing loci, expressed
Os07g47990	2	peroxidase precursor, putative, expressed
Os07g48020	2	peroxidase precursor, putative, expressed
Os07g48030	2	peroxidase precursor, putative, expressed
Os07g48280	2	expressed protein
Os08g37660	2	plastocyanin-like domain containing protein, putative, expressed
Os08g42030	2	peroxidase precursor, putative, expressed
Os09g30490	2	EF hand family protein, expressed
Os11g11960	2	disease resistance protein RPM1, putative, expressed
Os12g36880	2	pathogenesis-related Bet v I family protein, putative, expressed (PR-10)
Os01g34450	1	expressed protein
Os01g50410	1	STE_MEKK_ste11_MAP3K.6 - STE kinases
Os01g57740	1	expressed protein
Os01g67820	1	exo70 exocyst complex subunit domain containing protein, expressed
Os02g08440	1	OsWRKY71
Os02g33600	1	VQ domain containing protein, putative
Os02g43820	1	AP2 domain containing protein, expressed
Os02g56370	1	OsWAK20 - OsWAK receptor-like protein kinase, expressed
Os02g56700	1	dehydrogenase, putative, expressed (cinnamoyl CoA reductase)
Os03g01740	1	expressed protein
Os03g04070	1	no apical meristem protein, putative, expressed
Os03g44380	1	9-cis-epoxycarotenoid dioxygenase 1, chloroplast precursor, putative, expressed
Os03g50280	1	GLTP domain containing protein, putative, expressed
Os03g50410	1	lipase family protein
Os03g55180	1	DUF1336 domain containing protein, expressed
Os03g55800	1	cytochrome P450, putative, expressed

Os04g34140	1	U-box protein CMPG1, putative, expressed
Os05g08860	1	expressed protein
Os05g46830	1	proline-rich protein, putative, expressed
Os06g09310	1	zinc finger, C3HC4 type domain containing protein, expressed
Os07g32940	1	hypothetical protein
Os07g48060	1	peroxidase precursor, putative, expressed
Os07g48770	1	serine hydrolase domain containing protein, expressed
Os08g40690	1	glycosyl hydrolase, putative, expressed (chitinase)
Os11g02369	1	LTPL7 - Protease inhibitor/seed storage/LTP family protein precursor, expressed (PR-14)
Os11g09010	1	lipase, putative, expressed
Os12g02310	1	LTPL11 - Protease inhibitor/seed storage/LTP family protein precursor, expressed (PR-14)
