# Supplementary material for: The transcriptional network of WRKY53 in cereals links oxidative responses to biotic and abiotic stress inputs
Source: Funct Integr Genomics. 2014 Apr 29;14(2):351–62. doi: 10.1007/s10142-014-0374-3 (PMC4059961; doi:10.1007/s10142-014-0374-3)
Supplement: Supplementary file 6 — Primers used for SYBR Green I-based electrophoretic mobility shift assay. (PDF 23 kb) [file 10142_2014_374_MOESM6_ESM.pdf]

Table S3. Primers used for SYBR Green I-based electrophoretic mobility shift assay

Locus ID	Annotation	Primer sequences	
LOC_Os11g47600	*Chitinase-2*	Forward:<br>Reverse:	TTCTGGGGCGCGTGGAGGACGT<br>AGATGATGACGGTGGTGTAGAG
LOC_Os01g02300	*ORK10* kinase	Forward:<br>Reverse:	AGTTACCTTCGATAGGCCAAG<br>GGGAAGATTATTCCACAGTGGA
LOC_Os07g48050	*POC1* peroxidase	Forward:<br>Reverse:	CATGCCCACGTATGTGATAG<br>CTCTGTACTTCTCCAGCAGAGAC
LOC_Os07g48040	*POX5.1* peroxidase	Forward:<br>Reverse:	CCATGTTTATCCATCATATAA<br>ATATTCGGGAGTTATGTCTTC
