# Supplementary material for: The transcriptional network of WRKY53 in cereals links oxidative responses to biotic and abiotic stress inputs
Source: Funct Integr Genomics. 2014 Apr 29;14(2):351–62. doi: 10.1007/s10142-014-0374-3 (PMC4059961; doi:10.1007/s10142-014-0374-3)
Supplement: Supplementary file 2 — WRKY53 coexpression networks determined in silico. (a) RiceNet CytoScape probabilistic functional gene network indicating the presence of 36 loci coexpressed with LOC_Os05g27730, encoding OsWRKY53. (b) MSU Rice Genome Annotation Project Gene Coexpression Analysis. A total of 62 loci out of 1,161 in the M. oryzae infection-induced dataset were coregulated with LOC_Os05g27730 at a correlation >0.99, indicated in red. (PDF 98 kb) [file 10142_2014_374_MOESM2_ESM.pdf]

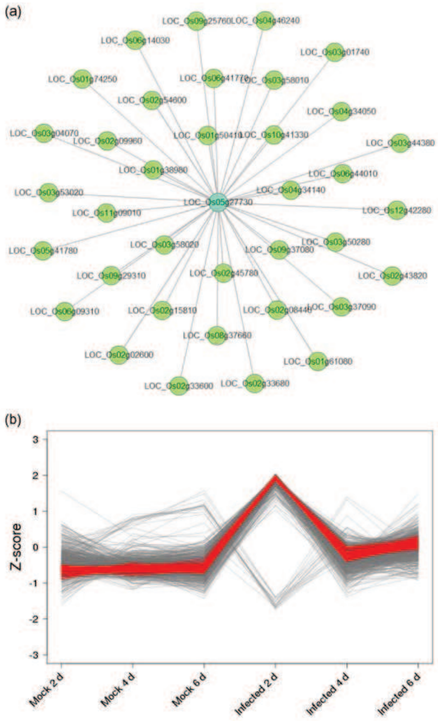

Figure S2. *WRKY53* coexpression networks determined *in silico*. (a) RiceNet CytoScape probabilistic functional gene network indicating the presence of 36 loci coexpressed with LOC_Os05g27730, encoding *OsWRKY53*. (b) MSU Rice Genome Annotation Project Gene Coexpression Analysis. A total of 62 loci out of 1,161 in the *M. oryzae* infection-induced dataset were coregulated with LOC_Os05g27730 at a correlation > 0.99, indicated in red.
